# A 60 year wave hindcast dataset in the Caribbean Sea

**DOI:** 10.1016/j.dib.2021.107153

**Published:** 2021-05-18

**Authors:** Andrés F. Orejarena-Rondón, Alejandro Orfila, Juan C. Restrepo, Isabel M. Ramos, Ismael Hernandez-Carrasco

**Affiliations:** a Grupo de Investigación en Geociencias GEO4, Departamento de Física y Geociencias, Universidad del Norte, km 5, Vía Puerto Colombia 081007, Colombia; b Instituto Mediterráneo de Estudios Avanzados (CSIC-UIB), Miquel Marqués, 21, Esporles 07190, Spain; c Balearic Islands Coastal Observing and Forecasting System (SOCIB), Palma de Mallorca 07021, Spain

**Keywords:** Hindcast, Waves, Caribbean, SWAN

## Abstract

This article presents a 60 years wave hindcast from 1958 to 2017, covering the Colombian Caribbean basin. Each output consists on 6-hour field of significant wave height $H_{s}$, mean wave period $T_{m-01}$, $T_{\text{mm}-10}$ and mean direction $\theta_{m}$ with a resolution of 11.8 km × 11.4 km. The simulation was performed using SWAN model forced with JRA-55 wind fields. Model data is validated against NOAA buoy 42058 located in the central Caribbean. The resolution and time spam of this database allows to perform either coastal engineering projects as well as to perform research in seasonal and interannual wave climate variability including large return periods to evaluate coastal vulnerability.

**Specifications Table**

**Table:** 

Subject	Ocean and Maritime Engineering
Specific subject area	Wave Hindcast
Type of data	Arrays
How data were acquired	SWAN Model
Data format	Raw
Parameters for data collection	The wave data ($H_{s}$, $T_{m-01}$, $T_{\text{mm}-10}$ and $\theta_{m}$) are obtained from a SWAN implementation [1]. The model is forced with 60 years of JRA-55 wind reanalysis. Simulated waves are validated against *in-situ* NOAA wave buoy.
Description of data collection	The wave parameters are provided every 6 h in yearly netCDF files, from January, 1st 1958 to December,31st 2017. Each file contains $H_{s}$, $\theta_{m}$, $T_{m-01}$, $T_{\text{mm}-10}$ latitude, longitude and time in 229 x 101 nodes covering the southern Caribbean basin. Each file is 1GB size.
Data source location	Instituto Mediterráneo de Estudios Avanzados City/Town/Region: Esporles Country:Spain
Data accessibility	Repository name: TIC IMEDEA NIMBUS in DIGITAL.CSIC Data identification number: 10.20350/digitalCSIC/13855 Direct URL to DIGITAL.CSIC: https://digital.csic.es/handle/10261/239546 Direct URL to data: https://nimbus.imedea.uib-csic.es/s/JEaPEeeQNLPFJ3S
	Direct URL to SWAN code: http://swanmodel.sourceforge.net/

## Value of the Data

• This database addresses the lack of information of wave parameters in the Caribbean basin. Database covers 60 years of wave fields suitable for scientific and engineering purposes.
• Data is addressed to climate scientists, ocean engineers, coastal managers and public administrations.
• This database is suited to (i) study of coastal vulnerability [2]; (ii) analysis of marine hazards through return periods [3]; (iii) marine energy analysis [4]; (iv) climate variability at multiple scales [5]; (v) works in coastal defense [6]; (vi) beach management and restoration [7] ; (vii) coral-reef protection [8].

## 1 Data Description

**Fig. 1:**
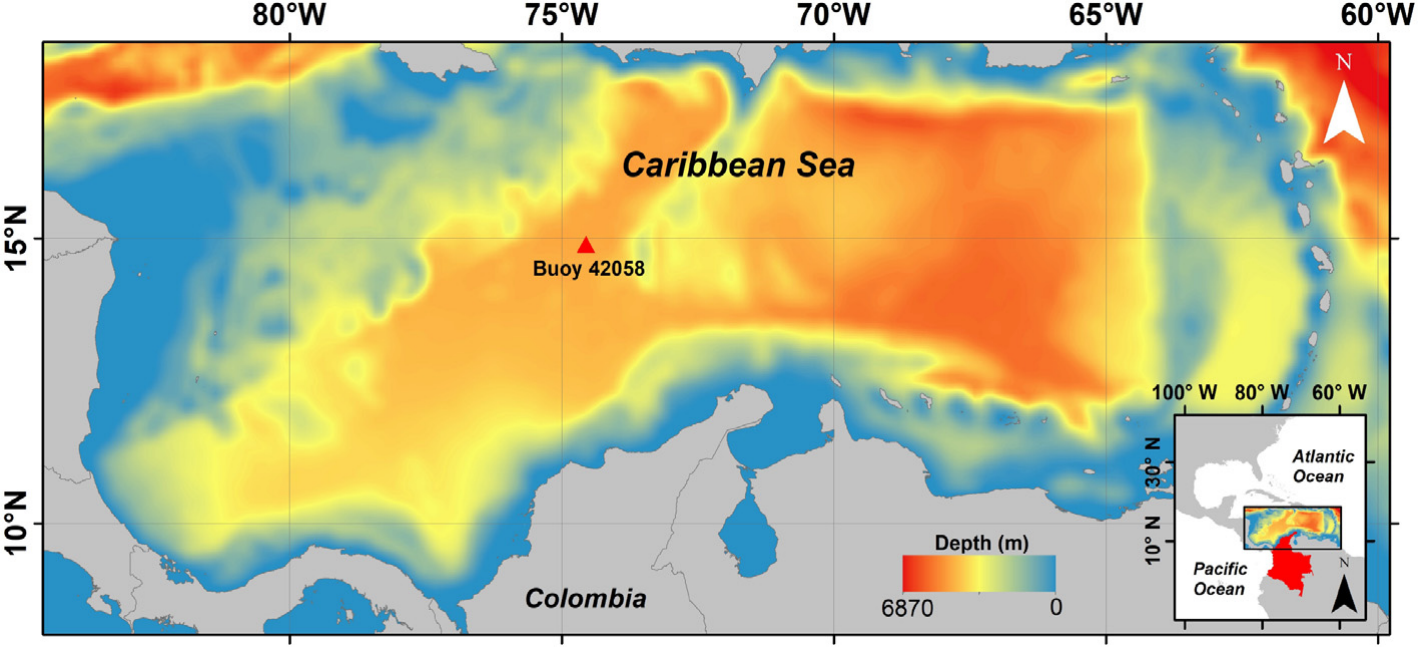
Geographycal domain of the hindcast. The red triangle indicate the location of NOAA 42058 used for model validation and the colorbar the bathymetry. (For interpretation of the references to color in this figure legend, the reader is referred to the web version of this article.)

Each file contains significant wave height $H_{s}$, mean wave period $T_{m-01}$ $T_{\text{mm}-10}$, mean direction $\theta_{m}$, latitude, longitude and time for a specific year. Each file in NetCDF format is around 1GB size containing the above data every 6 h at 00 h, 06 h, 12 h and 18 h. Data cover from January, 1st 1958 to December, 31st 2017 on a 229×101 mesh nodes with a resolution of 11.8 km × 11.4 km. Bottom left corner coordinates are −84.5219${}^{\circ }$ W; 8.0922${}^{\circ }$ N (Fig. 1).

## 2 Experimental Design, Materials and Methods

**Fig. 2:**
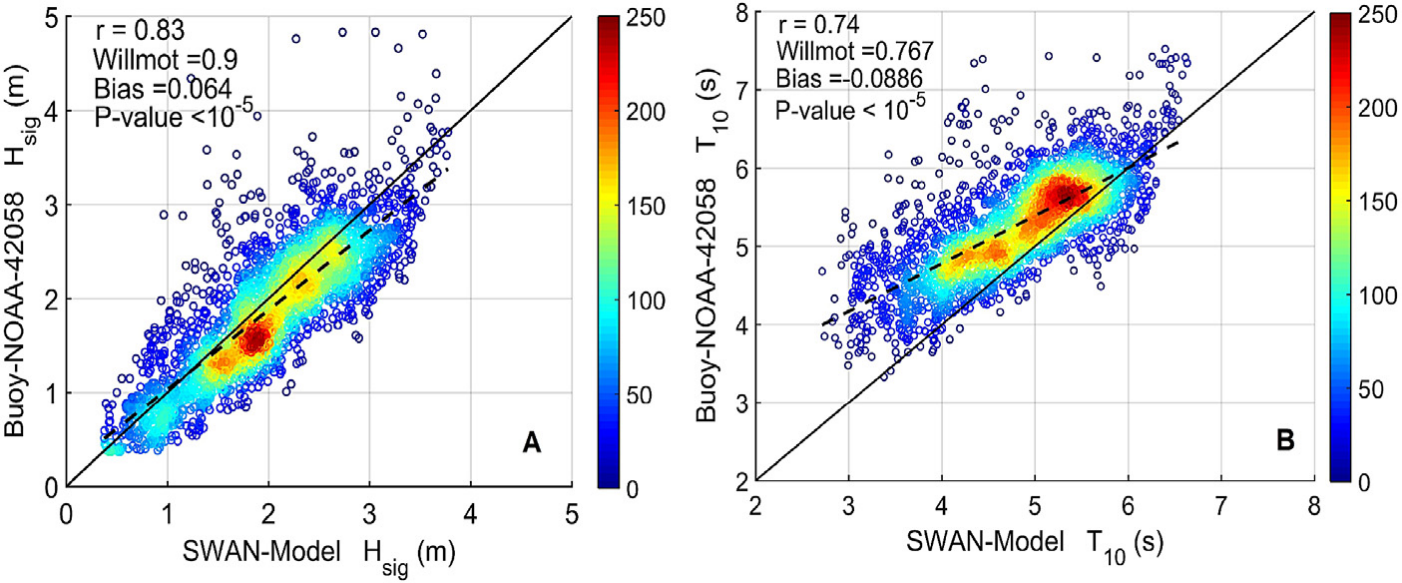
Validation of $H_{s}$ (A) and $T_{m-01}$ (B) in the Central Caribbean (Buoy 42058), with their respective dispersion diagrams and associated statistics. Color scale of dispersion diagrams corresponds to associated density of data used for validation. The linear regression is represented by the dashed line. (For interpretation of the references to color in this figure legend, the reader is referred to the web version of this article.)

SWAN is a third-generation wave model, developed at Delft University of Technology, that computes random, short-crested wind-generated waves in coastal regions and inland waters. Model was forced with JRA-55 wind reanalysis from 1958 to 2017 covering the Caribbean basin. Nonlinear deep-water interactions follow the Webb-Resio-Tracy method and wave growth due to wind has been configured as exponential, following the formulation of [9]. Other relevant processes, such as whitecapping energy dissipation, wave breaking and bottom friction have been included in the simulations. For the validation, the parameters of whitecapping were adjusted; the rate of whitecapping dissipation (Cd s) was set as 3.18·10−5 and the value of the wave steepness for the Pierson-Moskowitz spectrum (ˆs2PM) as 5.02·10−3. Several testshow that adjustment of other parameters do not provide further improvement when comparing the simulations with observed data. Time step was set as 30 min and $H_{s}$, $T_{m-01}$, and $\theta_{m}$ recorded every 6 h. $H_{s}$ and $T_{-10}$ was validated using NOAA buoy # 42058 (red triangle in Fig. 1. Scatter plot between NOAA buoy and closest model grid point for $H_{s}$ and $T_{m-01}$ are shown in Fig. 2. The domain of the wave model covers 2600 km × 1175 km on a 229 × 101 mesh. The bathymetry was obtained from GEBCO bathymetry and nautical charts from the Colombian Hydrographic service (DIMAR). Wind forcing was downloaded from https://rda.ucar.edu/datasets/ds628.0/index.html#!access. Wind forcing consists in 10m-height (u,v) wind components on 22×47 grid points with 60.8 km×60.7 km resolution every 6 h. The bottom left coordinates for the wind fields are −84.3746${}^{\circ }$ W; 7.5819${}^{\circ }$ N.

## CRediT Author Statement

**Andrs F. Orejarena-Rondn:** Conceptualization, validation, writing - original draft, writingreview & editing, Formal analysis; **Alejandro Orfila:** Conceptualization, Writing - review & editing, Supervision, Formal analysis; **Juan Camilo Restrepo:** Conceptualization, Supervision; **Isabel Maria Ramos:** Validation, Writing - review & editing; **Ismael Hernandez-Carrasco:** Conceptualization, Writing - review & editing.

## Declaration of Competing Interest

The authors declare that they have no known competing financial interests or personal relationships that could have appeared to influence the work reported in this paper. AO is member of the editorial board of Ocean Dynamics, Ocean Sciences and Frontiers in Marine Sciences.
