# Corrigendum to ‘A 60 year wave hindcast dataset in the Caribbean Sea’ Data in Brief, 37 (2021)/ 107153

**DOI:** 10.1016/j.dib.2021.107561

**Published:** 2021-11-08

**Authors:** Andrés F. Orejarena-Rondón, Alejandro Orfila, Juan C. Restrepo, Isabel M. Ramos, Ismael Hernandez-Carrasco

**Affiliations:** a Departamento de Física y Geociencias, Grupo de Investigación en Geociencias GEO4, Universidad del Norte, km 5, Vía Puerto Colombia, 081007, Colombia; b Instituto Mediterráneo de Estudios Avanzados (CSIC-UIB), Miquel Marqués, 21, Esporles 07190, Spain; c Balearic Islands Coastal Observing and Forecasting System (SOCIB), Palma de Mallorca 07021, Spain

The authors regret to acknowledge financial support from Ministerio de Ciencia e Innovación (MCI), Agencia Estatal de Investigación (AEI) and Fondo Europeo de Desarrollo Regional (FEDER) through MOCCA Project (RTI2018-093941-B-C31).

The authors would like to apologise for any inconvenience caused.

